# Clustering Approach for Detecting Multiple Types of Adversarial Examples

**DOI:** 10.3390/s22103826

**Published:** 2022-05-18

**Authors:** Seok-Hwan Choi, Tae-u Bahk, Sungyong Ahn, Yoon-Ho Choi

**Affiliations:** 1 School of Computer Science and Engineering, Pusan National University, Busan 46241, Korea; daniailsh@pusan.ac.kr (S.-H.C.); sungyong.ahn@pusan.ac.kr (S.A.); 2 Korea Apparel Testing & Research Institute, Seoul 02579, Korea; tu.bahk@pusan.ac.kr

**Keywords:** adversarial examples, adversarial perturbation, deep neural networks (DNNs), security, clustering

## Abstract

With intentional feature perturbations to a deep learning model, the adversary generates an adversarial example to deceive the deep learning model. As an adversarial example has recently been considered in the most severe problem of deep learning technology, its defense methods have been actively studied. Such effective defense methods against adversarial examples are categorized into one of the three architectures: (1) model retraining architecture; (2) input transformation architecture; and (3) adversarial example detection architecture. Especially, defense methods using adversarial example detection architecture have been actively studied. This is because defense methods using adversarial example detection architecture do not make wrong decisions for the legitimate input data while others do. In this paper, we note that current defense methods using adversarial example detection architecture can classify the input data into only either a legitimate one or an adversarial one. That is, the current defense methods using adversarial example detection architecture can only detect the adversarial examples and cannot classify the input data into multiple classes of data, i.e., legitimate input data and various types of adversarial examples. To classify the input data into multiple classes of data while increasing the accuracy of the clustering model, we propose an advanced defense method using adversarial example detection architecture, which extracts the key features from the input data and feeds the extracted features into a clustering model. From the experimental results under various application datasets, we show that the proposed method can detect the adversarial examples while classifying the types of adversarial examples. We also show that the accuracy of the proposed method outperforms the accuracy of recent defense methods using adversarial example detection architecture.

## 1. Introduction

As a core part of current real-world applications, deep learning technology has been widely applied not only in various fields but also even in security sensitive fields such as self-driving cars [1], malware classification [2], and face recognition system [3]. However, many studies showed that deep learning technology is very vulnerable to adversarial examples, which deceive the deep learning model by adding human-imperceptible perturbations to input data. As a representative example, Hussain, S. et al. recently showed that even deep learning-based defense method, such as DeepFake detector, can be bypassed by adversarial examples at WACV conference in 2021 [4].

To defend against adversarial examples, many defense methods have been proposed. Based on the types of defense architectures, such defense methods are commonly classified into three categories, i.e., model retraining architecture, input transformation architecture and adversarial example detection architecture. The model retraining architecture makes deep learning model more robust before adversaries generate adversarial examples by retraining deep learning models or training with new models [5,6,7]. The model retraining architecture is known to be effective for adversarial examples with large perturbation such as Fast Gradient Sign Method (FGSM) [8] and Basic Iterative Method (BIM) [9]. On the other hand, the input transformation architecture reduces the perturbation of adversarial examples by transforming the input data before feeding into the deep learning model [10,11,12]. The input transformation architecture can provide robustness with low memory usage and computation costs against adversarial examples with small perturbation such as DeepFool [13] and C&W [14]. Even though previous defense methods belonging to two architectures provided good robustness against adversarial examples, the classification accuracy decreases when predicting the legitimate input data because such two architectures affect the legitimate input data or deep learning model. Specifically, for the defense methods using model retraining architecture, the classification accuracy of deep learning model decreases because they train or re-train the model with noise contained dataset. For the defense methods using input transformation architecture, the classification accuracy of deep learning model decreases because they transform not only adversarial examples but also legitimate input data.

**Figure 1:**
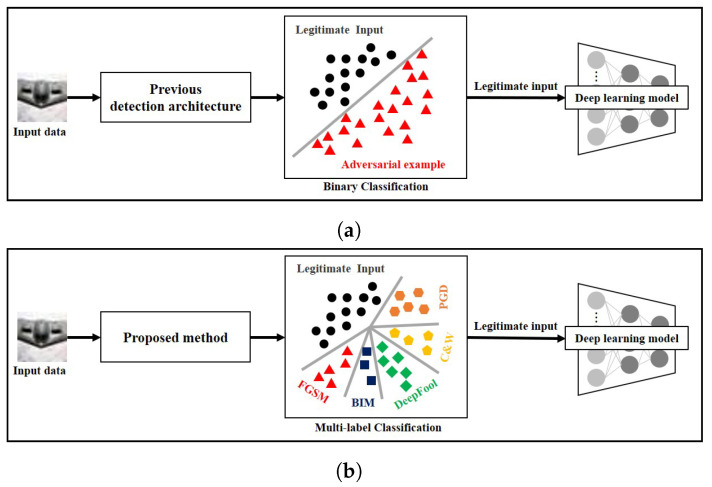
Difference between the previous defense methods using adversarial example detection architecture and the proposed method. (**a**) Previous defense methods using adversarial example detection architecture. (**b**) Proposed method.

To address this problem, defense methods using adversarial example detection architecture have been actively studied [15,16,17,18]. As shown in Figure 1a, the defense methods using adversarial example detection architecture classify the input data into either legitimate one or adversarial one. To achieve this, the defense methods using adversarial example detection architecture compute the probability that the input data is an adversarial example or compare the distribution between adversarial examples and legitimate input data. However, the defense methods using adversarial example detection architecture do not classify the input data into multiple classes of data, i.e., legitimate input data and various types of adversarial examples. Classifying the input data into multiple classes of data is important for many researchers and security experts to analyze the characteristics of each adversarial example. Based on these characteristics, they can establish an appropriate defense method or design a new defense method for unknown adversarial examples.

In this paper, to overcome the limitation of the defense methods using adversarial example detection architecture, we propose an advanced defense method using adversarial example detection architecture. Different from the previous defense methods using adversarial example detection architecture, the proposed method can detect adversarial examples while classifying the types of adversarial examples as shown in Figure 1b. Detecting adversarial examples means that the proposed method can be used as a stand-alone defense method against adversarial examples. Additionally, classifying various types of adversarial examples means that the proposed method can be utilized as an analysis tool to analyze the characteristics of adversarial examples.

A high-level operations of the proposed method is as follow: first, given an input data (a legitimate input data or an adversarial example), the proposed method extracts adversarial perturbations using denoising techniques, such as a binary filter and median smoothing filter. Second, the proposed method reduces the dimensionality of the extracted adversarial perturbations. Finally, the proposed method uses a clustering model as a detector. Specifically, the clustering model classifies the reduced features of input data into multiple classes, i.e., legitimate input data and various types of adversarial examples.

From the experimental results under various datasets collected from diverse applications, we show that only the proposed method can classify the types of the adversarial examples. We also show that the accuracy of the proposed method outperforms the accuracy of recent defense methods using adversarial example detection architecture, e.g., Carrara et al.’s method [15] and Feature Squeezing [16]. Such results can be actively used to design new defense architecture, analyze the characteristics of adversarial examples, and improve the existing defense methods. For example, we can analyze the characteristics of adversarial examples from the clustering results and selectively apply the defense methods suitable for each characteristic.

The contributions are summarized as follows:

- We propose a novel defense method using adversarial example detection architecture. Different from the current defense methods using adversarial example detection architecture, which can classify the input data into only either legitimate one or adversarial one, the proposed method classifies the input data into multiple classes of data;
- To the best of our knowledge, the existing defense methods including model retraining architecture, input transformation architecture and adversarial example detection architecture use the adversarial example itself for analysis or detection. Thus, this is the first work which approximates the adversarial perturbation of each adversarial example and uses it to detect adversarial examples;
- From analysis results under various adversarial examples and application datasets, we show that the proposed method provides better accuracy than recent defense methods using adversarial example detection architecture, e.g., Carrara et al.’s method [15] and Feature Squeezing [16].

The rest of the paper is organized as follows. In Section 2, we overview the well-known adversarial examples and the recent defense methods using adversarial example detection architecture. In Section 3, we describe the overall operation and the details of the proposed method. In Section 4, we verify the effectiveness of the proposed method from various experimental results under different adversarial examples, different datasets and so on. Finally, we conclude this paper in Section 6.

## 2. Preliminaries and Related Works

### 2.1. Adversarial Examples

In this section, we summarize the characteristics of five well-known adversarial examples, which are used for experimental evaluation in this paper [5,8,9,13,19].

Goodfellow et al. proposed a non-iterative-based fast method to generate adversarial examples, called Fast Gradient Sign Method (FGSM) [8]. To generate adversarial examples, FGSM increases the loss of the deep learning model using the sign of the gradient. As a iterative-based fast method to generate adversarial examples, Basic Iterative Method (BIM) was introduced by Kurakin et al. [9]. Different from FGSM which performs only one gradient update, BIM performs iterative gradient updates for fine optimization. Projected Gradient Descent (PGD) is another iterative-based method introduced by Madry et al. [5]. To perform fine optimization efficiently, PGD performs iterative gradient updates from randomly selected an initial point.

To generate adversarial examples with minimal perturbations, Moosavi-Dezfooli et al. introduced a DeepFool which performs an iterative linearization of the deep learning model [13]. In each iteration, DeepFool finds the nearest decision boundary from an input *X*, and updates the adversarial perturbation to reach that decision boundary. To increase attack success rate while generating adversarial examples with minimum perturbations, Carlini and Wagner introduced three adversarial examples based on various distance metrics such as $L_{0}$, $L_{1}$ and $L_{2}$ norm [19]. In this paper, we consider the $L_{2}$ type of C&W as a representative method because it is most frequently mentioned in other works [14].

### 2.2. Defense Methods Using Adversarial Example Detection Architecture

In this section, we summarize some recent defense methods using adversarial example detection architecture.

Dathathri et al. [20] proposed a signature-based detection method against adversarial examples. To detect adversarial examples, they generate a NeuralFingerprinting (NFP) from the input data and checked a behavioral consistency of the target model for the NFP. Fidel et al. [21] also used the signature, called the XAI signature, to detect adversarial examples. To generate a XAI signature for the input data, they used Shapley Additive Explanations (SHAP) values computed for the internal layers of a target model.

To provide the robustness to deep learning models against adversarial examples, Metzen et al. [22] proposed a subnetwork-based detector which classifies the input data into either legitimate input or adversarial examples. They added the binary detector subnetwork to one of the middle layers of the target deep learning model and trained it to learn the probability that the input data is an adversarial example. Jiajun Lu et al. [23] also proposed a subnetwork-based detector, called SafetyNet. Different from the Metzen et al.’s method which the binary detector subnetwork is located in one of the middle layers of the target deep learning model, the detector in SafetyNet is located in the last layer of the target deep learning model.

Santhanam et al. [24] proposed a GAN-based defense method, called cowboy, for the detection and purification of adversarial examples. They argued that adversarial examples lie outside of the data distribution. The discriminator network in cowboy detects the adversarial examples and the generator network purifies the detected adversarial examples by projecting back to the data distribution. Yu et al. [25] also proposed a GAN-based detection method using Conditional Generative Adversarial Networks (CGANs). The discriminator network of Yu et al.’s method is not used detect adversarial examples directly, but to improve the performance of generator network.

Xu et al. [16] proposed a simple detection method against adversarial examples, called Feature Squeezing. Feature Squeezing consists of a squeezer and a detector. The squeezer transforms the input data into squeezed input to reduce the magnitude of adversarial perturbations. The detector compares the prediction on the input with the prediction on squeezed inputs to detect adversarial examples. Zheng et al. [26] proposed a part-based feature squeezing, which is specialized in person re-identification (ReID) field, by extending Feature Squeezing. Different from the Feature Squeezing, which uses a squeezer to the entire image, Zheng et al.’s method divides the image into several parts and uses a squeezer to each part. Abusnaina et al. [17] proposed a graph-based detection method against adversarial examples. To detect adversarial examples, Abusnaina et al.’s method constructs a Latent Neighborhood Graph (LNG) around an input data and uses Graph Neural Networks (GNNs) as a detector.

Carrara et al. [15] proposed a detection method against adversarial examples using Long Short-Term Memory (LSTM) models. They argued that intermediate features extracted from all layers of the target deep learning model must be used to determine the tendency or direction of adversarial examples. Since the number of intermediate features extracted from all layers is very large, they reduced the dimensionality of intermediate features by using global average pooling. After reducing the dimensionality, they generated the sequential vector using distance metric-based embedding technique and trained the LSTM-based detector. Wang et al. [18] proposed a model-agnostic approach to detect adversarial examples. They argued that there is an intrinsic difference of the logit semantic distribution between legitimate input data and adversarial examples. To capture differences in the distribution of logits sequences, they used the LSTM network as a detector. We summarize the characteristics of representative defense methods using adversarial example detection architecture in Table 1 for comparison.

## 3. Proposed Method

In this section, we overview the operation of the proposed method in details. We also describe each operation of the proposed method and support it with specific examples.

### 3.1. Overall Operation

To detect adversarial examples while classifying the types of adversarial examples, the proposed method follows three steps: (1) Adversarial Perturbation Extraction; (2) Dimensionality Reduction; and (3) Clustering.

**Figure 2:**
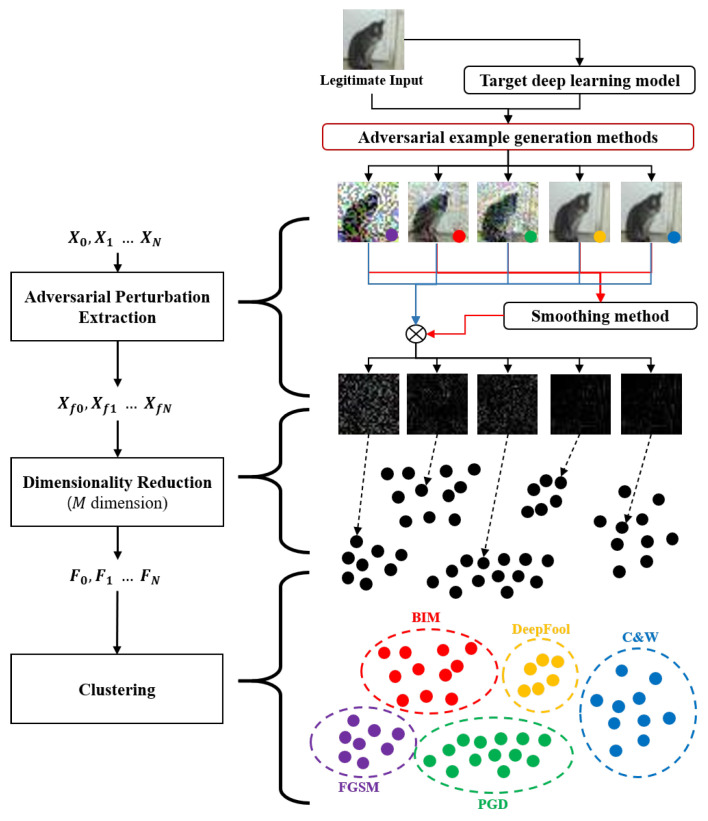
Overall operation of the proposed method.

In Figure 2, a high-level illustration of the proposed method is described. In Adversarial Perturbation Extraction step, the proposed method extracts the perturbations, which are added by the adversary from the input data. Since it is not known whether an adversarial example has happened or which adversarial example has performed, the proposed method first applies the denoising techniques to the input data to obtain the denoised input. Then, the proposed method calculates the perturbations between the input data and the denoised input. In Dimensionality Reduction step, the proposed method extracts the key features from the perturbations, which are extracted in Adversarial Perturbation Extraction step, by reducing the feature dimensionality. In the Clustering step, the proposed method feeds the extracted features into the clustering model to detect adversarial examples while classifying the types of adversarial examples. Further details on each step are provided in the subsequent sections.

### 3.2. Adversarial Perturbation Extraction

Different from the previous defense methods using adversarial example detection architecture which use the adversarial examples itself for detecting, the proposed method uses the adversarial perturbation which was added to input data by the adversary. However, it is impossible to get the adversarial perturbation directly from the adversarial example because it is not known whether an adversarial example has happened to input data or which adversarial example has performed on input data. Thus, the adversarial perturbation is approximated by using two denoising techniques, binary filter and median smoothing filter, which have shown good performance in other works [14,16].

**Figure 3:**
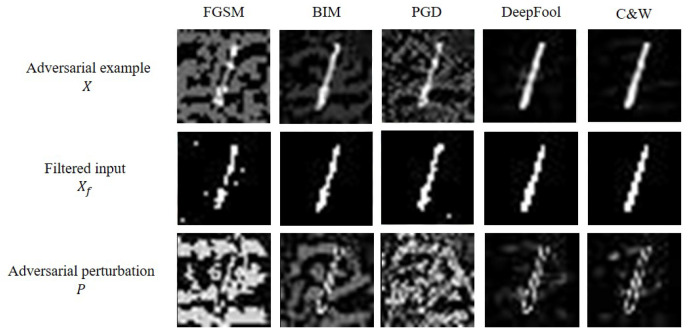
Five extracted adversarial perturbation from adversarial examples in MNIST test dataset, where each perturbation is denoised using a binary filter.

For a gray-scale image dataset such as the MNIST dataset [27], the proposed method approximates the adversarial perturbation by using the binary filter which can reduce the redundancy while keeping the key features of gray-scale images. Here, since the binary filter perceives adversarial perturbation as redundancy, it can remove adversarial perturbation well. Specifically, given an input data *X*, the binary filter reduces the bit-depth of input data *X* to *i*-bit depth (1≤i≤7) as follow:

(1) $$\begin{array}{c} x=round\left(x\times 2^{i-1}\right)/\;2^{i-1},\;\;\forall x\in X, \end{array}$$

where round(·) is a round function, which rounds to the nearest integer. We set *i* of the binary filter into 1, which showed the best performance for the MNIST dataset [16,28]. Some examples of a binary filter for the MNIST dataset under various adversarial examples are shown in Figure 3.

**Figure 4:**
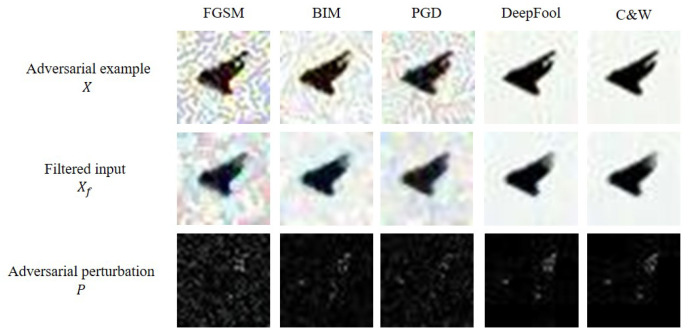
Five extracted adversarial perturbations from adversarial examples in CIFAR-10 test dataset, where each perturbation is denoised using a median filter.

For color image dataset such as CIFAR-10 dataset [29], the proposed method approximates the adversarial perturbation by the median smoothing filter because it can remove adversarial perturbation well while preserving the sharpness of edges for color images. To remove the adversarial perturbation, the median smoothing filter reduces variation among pixels. Specifically, given an input data *X*, the filtered input $X_{f}$ can be obtained as follows:

(2) $$\begin{array}{c} X_{f}\left[u,v\right]=median\left\{X\left[u+i,v+j\right]\;|\;-1\leq \left(i,j\right)\leq 1\right\}, \end{array}$$

where [u,v] is the position of input data *X* and median{·} is a median function, which returns a median value among values in a range. Some examples of median smoothing filter for CIFAR-10 dataset under various adversarial examples are shown in Figure 4. From “Filtered input” in Figure 3 and Figure 4, it is observed that such denoising techniques effectively work regardless of the types of adversarial examples.

After getting the filtered input from two denoising techniques, the adversarial perturbation is approximated by calculating arithmetic subtraction operations between the input data and the filtered input. By using the extracted adversarial perturbations rather than the adversarial examples, the proposed method can consider only the key features of the adversarial examples, without considering any key features of legitimate input data.

### 3.3. Dimensionality Reduction

Using the extracted perturbation directly to classify the types of adversarial examples is not efficient because the perturbation contains unnecessary values. For example, as shown in Figure 4, most pixels of the extracted adversarial perturbation are black in color and it means that such pixels do not affect classification of the types of adversarial examples. Thus, the proposed method performs the dimensionality reduction to extract the key features from the adversarial perturbation. Specifically, the proposed method uses two dimensionality reduction methods, i.e., Principal Component Analysis (PCA) and Linear Discriminant Analysis (LDA).

PCA is an unsupervised dimensionality reduction method which maximizes the variance between data without considering the class separation. By finding a few orthogonal linear combinations, which are called Principal Components of multivariate data with the largest variance, and PCA can reduce the dimension of the data. As one of the most commonly used dimensionality reduction methods in various fields, PCA has shown good feature extraction performance even for image data.

On the other hand, LDA is a supervised dimensionality reduction method which maximizes the variance between samples in different classes while minimizing the variance between samples in the same class. Different from PCA, which deals with the data in its entirety and searches for vectors that best describe the data, LDA deals directly with discrimination between classes and searches for vectors that best describe the class. LDA can reduce the dimensionality without loss of information. The detailed impact of each dimensionality reduction method on clustering performance is described in Section 4.2.2.

### 3.4. Clustering

The goal of the proposed method is not only to detect adversarial examples but also to classify the types of adversarial examples. To achieve this, the proposed method uses a clustering model as a detector. When the extracted features *F* and the ground truth label *l* are given, the objective function of the detector can be expressed into:

(3) $$\begin{array}{cc} \; & \max_{C(\cdot )}Pr\left(l^{\prime }=l\right)\\ s.t.\; & l^{\prime }\;=\;arg maxC\left(F\right), \end{array}$$

where C(·) is a clustering model. In this paper, the proposed method uses two clustering models, i.e., the k-means algorithm and the Density-based spatial clustering of applications with noise(DBSCAN) algorithm.

The k-means algorithm is one of the most well-known simplest clustering methods that has been used in a variety of application domains. To find clusters, the k-means algorithm groups and classifies data that are similar to each other in the entire dataset. The pseudo code of the k-means algorithm is illustrated in Algorithm 1. The k-means algorithm requires a parameter *k*, which represents the number of output clusters. *k* can be set according to the number of adversarial examples, which the defender wants to classify.   
**Algorithm 1 **Pseudo code of k-means algorithm
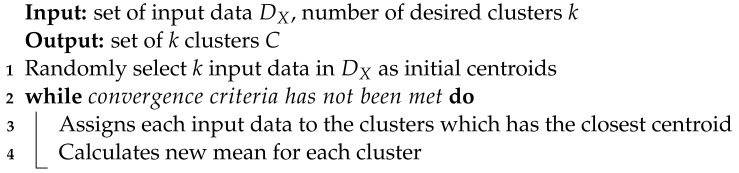


   The DBSCAN algorithm is a well-known density-based clustering method which is very effective when finding arbitrarily shaped clusters. As shown in Algorithm 2, the DBSCAN algorithm finds clusters by calculating the density of each data with neighboring data. The DBSCAN algorithm requires two parameters: (1) epsilon ($P_{eps}$), which represents how close points should be to each other to be contained in the same cluster; and (2) the minimum number of points ($P_{MinPts}$), which represents minimum number samples to be contained in one cluster. Different from the k-means algorithm, the DBSCAN algorithm does not need a parameter for the number of output clusters. In other words, the DBSCAN algorithm can learn the number of clusters automatically.   
**Algorithm 2 **Pseudo code of the DBSCAN algorithm
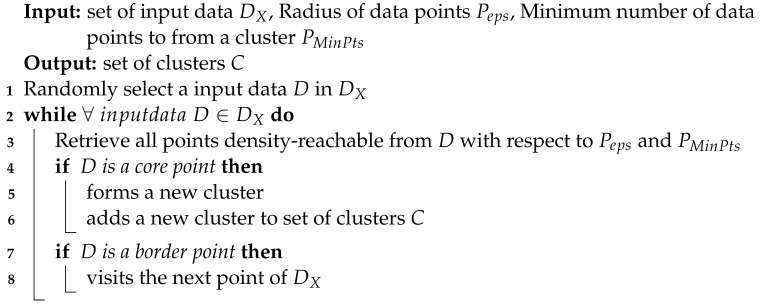


### 3.5. Operational Example

**Figure 5:**
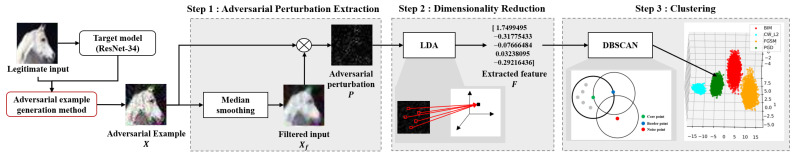
Operational example of the proposed method with LDA and the DBSCAN algorithm.

In this section, we show an example of how the proposed method works for the CIFAR-10 test dataset. Here, let us consider that a target model is the ResNet-34 model [30], and the proposed method uses LDA and the DBSCAN algorithm as a dimensionality reduction method and a clustering model, respectively. Let us consider PGD occurring to the horse sample. As shown in Figure 5, when the attacked horse sample is given as an input data, the proposed method extracts perturbation from the input data. Here, it is observed that most of the pixels have a value of 0 (black color) and only a few pixels have non zero values. Next, the proposed method extracts key features from the extracted adversarial perturbation by reducing the feature space. Specifically, the proposed method reduces the extracted adversarial perturbation of 32 × 32 × 3 dimensionality to a vector of 5 × 1 dimensionality. These extracted features of 5 × 1 dimensionality are used as an input to a clustering model. As a result, it is observed that the attacked horse sample by PGD is clustered in the same cluster with other PGD samples.

## 4. Evaluation Results

To show how efficient the proposed method is when classifying the types of adversarial examples, we measured the performance of the proposed method under various conditions including different adversarial examples.

### 4.1. Experimental Setup

In this section, we describe experimental environments including dataset, target model, evaluation metric, and so on.

Dataset: The experiments are conducted using the MNIST dataset [27] and the CIFAR-10 dataset [29]. The MNIST dataset is grayscale Hand-written digits dataset and consists of 60,000 training images and 10,000 testing images corresponding to 10 classes. CIFAR-10 is color image dataset and consists of 50,000 training images and 10,000 testing images corresponding to 10 classes. Five types of adversarial examples (FGSM, BIM, PGD, DeepFool, and C&W) are used to train the clustering model. Specifically, 9790 adversarial examples for each adversarial example type from the MNIST dataset are used to train the clustering model. For the CIFAR-10 dataset, 7189 adversarial examples for each adversarial example type are used to train the clustering model.

Target Model: Even though the proposed method is not directly affected by the target model, an adversary needs to have complete access to the parameter values used for training the target deep neural network model to generate adversarial examples. In this paper, one deep learning model per each benchmark dataset is used as the target model. For the MNIST dataset, a basic Convolutional Neural Network (CNN) model, which is commonly used in image classification areas, is used to a target model. Such CNN model consists of convolutional layer of 5 × 5, max pooling layer of 2 × 2, one fully connected layer for flattening, and the softmax function applied to the output layer. Each convolutional layer uses the ReLU function as an activation function. The output layer consists of 10 neurons, each of which represents an image label. The Adam optimization function is used to train the CNN model. The batch size, epoch, and learning rate are set to 128, 10, and 0.001, respectively. For the CIFAR-10 dataset, the proposed method uses a ResNet-34 model [30] as a target model. As with the CNN model, the Adam optimization function is used to train the ResNet-34 model. The batch size, epoch, and learning rate are set to 128, 20, and 0.001, respectively.

Attack Configuration: When generating adversarial examples for the MNIST dataset, the parameter values are set as follows: (1) 0.3 for the magnitude of perturbation (ϵ) in FGSM; (2) 20 and 0.3 for the number of iterations (*N*) and ϵ, respectively, in BIM; (3) 20 and 0.3 for the number of iterations (*N*) and ϵ, respectively, in PGD; (4) 50 and 0.02 for the maximum number of iterations and overshoot to prevent updates from vanishing, respectively, in DeepFool; and (5) 0 for the parameter to control the confidence value (κ) in C&W. On the other hand, when generating adversarial examples for the CIFAR-10 dataset, the parameter values are set into: (1) 0.2 for the magnitude of perturbation (ϵ) in FGSM; (2) 20 and 0.2 for the number of iterations (*N*) and ϵ, respectively, in BIM; (3) 20 and 0.2 for the number of iterations (*N*) and ϵ, respectively, in PGD; (4) 80 and 0.2 for the maximum number of iterations and overshoot to prevent updates from vanishing, respectively, in DeepFool; and (5) 0 for the parameter to control the confidence value (κ) in C&W. These parameter values are set following the recommended configuration values from the cleverhans library [31] and some representative works [14,16].

Evaluation Metric: To evaluate the performance of the proposed method under different types of dimensionality reduction methods and clustering methods, we measure the V-measure and its two components, i.e., Homogeneity and Completeness.

Here, Homogeneity is a measure of how well a clustering model matches each cluster to each type of adversarial examples. When an array of input data *X* and the ground truth label *l* are given, Homogeneity can be defined as:

(4) $$\begin{array}{c} h=1-H(l|X)/H(l), \end{array}$$

where H(·) and H(·|·) are the entropy and conditional entropy functions, respectively.

As a measure of how well all data points of each type of adversarial examples are contained in the same cluster, Completeness can be defined as:

(5) $$\begin{array}{c} c=1-H(X|l)/H(X). \end{array}$$

V-measure is a measure defined as the harmonic mean of Homogeneity and Completeness and can be defined as:

(6) $$\begin{array}{c} v=2(h\;\times \;c)/(h+c). \end{array}$$

Implementation Environment: The classification models are implemented using TensorFlow-gpu version 1.14.1 and Python version 3.6.9. Adversarial examples are generated by using the cleverhans software library [31], which provides standardized reference implementations of adversarial examples. For the efficient experiments, the performance is measured on the Ubuntu 18.04.3 LTS machine with kernel version 5.3.0-62-generic, 2.40 GHz CPU clock Intel Xeon CPU E5-2630 v3), GeForce GTX 2080 Ti, and 64 GB memory.

### 4.2. Experimental Analysis

#### 4.2.1. Ablation Analysis

To verify the effectiveness of each step of the proposed method, we conducted ablation analysis on each step of the proposed method. Specifically, we measured the clustering performances of the proposed method according to the combination of each step using three evaluation metrics, i.e., Homogeneity, Completeness, and V-measure. Here, we considered LDA and the k-means algorithm as the dimensionality reduction method and the clustering method, respectively. The experimental results are summarized in Table 2.

In Table 2, it is observed that adversarial examples are not suitable for classifying the types of adversarial examples. For example, while the proposed method using an adversarial example showed 0.249 and 0.128 for clustering performance on average under the MNIST dataset and the CIFAR-10 dataset, respectively, the proposed method using adversarial perturbations showed 0.864 and 0.701 for clustering performance on average. It is also observed that the proposed method showed a higher performance as it performs each step. For the MNIST dataset, while the proposed method that performed steps 1 and 3 showed 0.627 for the clustering performance on average, the proposed method that performed steps 1, 2 and 3 showed 0.864 for the clustering performance on average. For the CIFAR-10 dataset, while the proposed method that performed steps 1 and 3 showed 0.378 for the clustering performance on average, the proposed method that performed steps 1, 2 and 3 showed 0.701 for the clustering performance on average.

**Figure 6:**
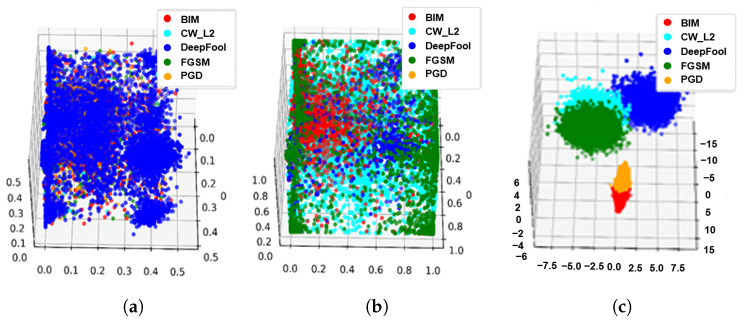
Visualized clustering results of the proposed method under the MNIST dataset. (**a**) Adversarial example + k-means (Step3). (**b**) Adversarial perturbation + k-means (Step 1 + Step 3). (**c**) Adversarial perturbation + LDA + k-means (Step 1 + Step 2 + Step 3).

**Figure 7:**
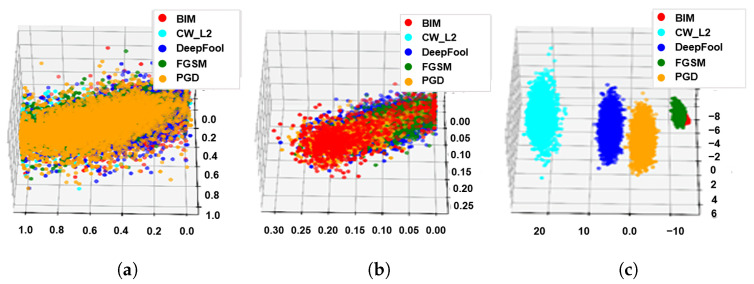
Visualized clustering results of the proposed method under the CIFAR-10 dataset. (**a**) Adversarial example + k-means (Step3). (**b**) Adversarial perturbation + k-means (Step 1 + Step 3). (**c**) Adversarial perturbation + LDA + k-means (Step 1 + Step 2 + Step 3).

In Figure 6 and Figure 7, we also show the visualized clustering results of the proposed method. As shown in Figure 6c and Figure 7c, the proposed method can classify the types of adversarial examples when it performed steps 1, 2 and 3.

#### 4.2.2. Influence of Dimensionality Reduction and Clustering Methods

To evaluate the proposed method under various combinations of dimensionality reduction methods and clustering methods, the clustering performances of the proposed method are measured using three evaluation metrics, i.e., Homogeneity, Completeness, and V-measure. To train the clustering model, five types of adversarial examples (FGSM, BIM, PGD, DeepFool, C&W) are used. Each type of adversarial example is generated using 1000 randomly selected datapoints from the MNIST test dataset and the CIFAR-10 test dataset, respectively. The *k* of k-means algorithm is set to 5, $P_{eps}$ of the DBSCAN algorithm is set to 0.878 for the MNIST dataset and 0.446 for the CIFAR-10 dataset, respectively.

In Table 3, it is observed that the different combination of dimensionality reduction methods and clustering methods showed the different influence on the clustering performance. From the ‘Average’ column in Table 3, it is observed that the proposed method showed the clustering performance from 0.764 to 0.865 under the MNIST dataset and from 0.513 to 0.716 under the CIFAR-10 dataset. Especially, the combination of PCA and the DBSCAN algorithm showed the lowest clustering performance on average under both MNIST and CIFAR-10 dataset. On the other hand, the combination of LDA and the DBSCAN algorithm showed the highest clustering performance on average.

It is also observed that LDA provides better feature extraction than PCA. For example, for the CIFAR-10 dataset, while the combination of PCA and the k-means algorithm and the combination of PCA and the DBSCAN algorithm showed 0.646 and 0.513 on average for the clustering performance respectively, the combination of LDA and the k-means algorithm and the combination of LDA and the DBSCAN algorithm showed 0.701 and 0.716 on average for the clustering performance respectively.

#### 4.2.3. Influence of the Number of Adversarial Examples Types

**Figure 8:**
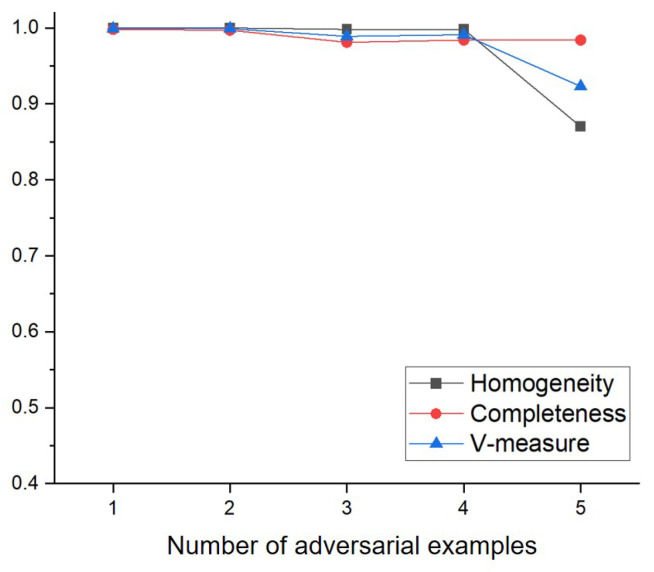
Clustering performance of the proposed method with the increase of the number of adversarial examples using MNIST dataset.

**Figure 9:**
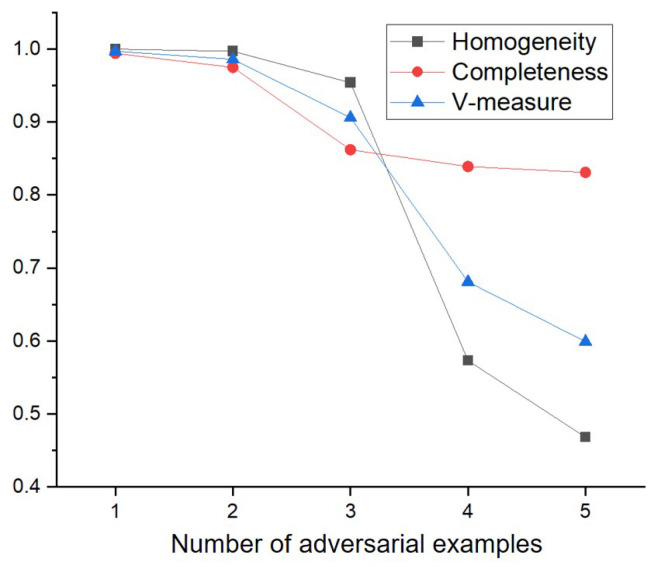
Clustering performance of proposed method with the increase of the number of adversarial examples using CIFAR-10 dataset.

To evaluate the clustering performance of the proposed method under the various number of adversarial examples, the experiments are conducted by gradually varying the number of adversarial examples. Specifically, the experiments are performed by adding 1000 of each of FGSM, C&W, BIM, PGD, and DeepFool in sequence to 1000 legitimate input data. The results for Homogeneity, Completeness and V-measure of the proposed method are shown in Figure 8 and Figure 9.

For the MNIST dataset, as shown in Figure 8, the proposed method maintained the clustering performance until the number of adversarial examples was four. Specifically, the proposed method showed 0.999, 0.999, 0.998 and 0.998 for the Homogeneity when the number of adversarial examples was 1, 2, 3 and 4, respectively. However, it is observed that when the number of adversarial examples was five, the clustering performance of the proposed method slightly decreased. For example, while the proposed method showed 0.991 for the V-measure when the number of adversarial examples was four, the proposed method showed 0.923 for the V-measure when the number of adversarial examples was five. It is also observed that the Completeness was maintained but the Homogeneity slightly decreased when the number of adversarial examples was five. It means that several instances of a particular adversarial example were included in a cluster of other adversarial examples.

For the CIFAR-10 dataset, as shown in Figure 9, the clustering performance of the proposed method began to decrease when the number of adversarial examples was higher than or equal to three. For example, while the proposed method showed 0.994 and 0.975 for the Completeness when the number of adversarial examples was 1, 2, respectively, the proposed method showed 0.862 for the Completeness when the number of adversarial examples was three. Especially, the Homogeneity and the V-measure of the proposed method significantly decreased when the number of adversarial examples was four. Specifically, the Homogeneity of the proposed method decreased from 0.954 to 0.573, and the V-measure of the proposed method decreased from 0.906 to 0.681. Even though the proposed method showed low Homogeneity and V-measure when the number of adversarial examples was higher than or equal to four, the proposed method showed high Completeness even when the number of adversarial examples increased. It means that most instances of each adversarial example are contained in the same cluster.

#### 4.2.4. Comparison to Other Defense Methods Using Adversarial Example Detection Architecture

To compare the proposed method with other recent defense methods using adversarial example detection architecture, the accuracy, precision, recall and F1-score of the proposed method are compared with those of Carrara et al.’s method [15] and Feature Squeezing [16]. Since the source codes of both methods are provided as open source, we selected them for comparison with the proposed method. For Carrara et al.’s method, we used Euclidean distance or cosine similarity as an embedding function and used Long Short-Term Memory (LSTM) and multi-layer perceptron network (MLP) as a detector. For Feature Squeezing, we used two squeezing methods, binary filter and median filter and used three distance metrics, $L_{1}$, $L_{2}$ and *K*-*L* diversity.

As observed from the “Accuracy” column to the “F1-score” column in Table 4, it is observed that Carrara et al.’s method works well with the CIFAR-10 dataset and Feature Squeezing works well with the MNIST dataset. For example, while Carrara et al.’s method showed the accuracy, precision, recall and F1-score to be as much as 70.56%, 66.49%, 78.11% and 72.40% on average for the MNIST dataset, Feature Squeezing showed the accuracy, precision, recall and F1-score as much as 94.52%, 95.27%, 93.65% and 94.2% on average. It is also observed that the proposed method showed a better binary classification performance on accuracy and F1-score than Carrara et al.’s method and Feature Squeezing. For example, while Carrara et al.’s method and Feature Squeezing showed an accuracy as much as 76.22% and 51.67% on average for the CIFAR-10 dataset, respectively, the proposed method showed an accuracy of as much as 83.32%.

In Table 5, it is observed that the proposed method can classify the types of adversarial examples. Here, since the recent defense methods did not provide the classification performance for multi-label, we only measured the classification performance of the proposed method. For the MNIST dataset, the proposed method showed accuracy, precision, recall and F1-score as much as 66.33%, 66.29%, 66.66% and 66.56%, respectively. For the CIFAR-10 dataset, the proposed method showed accuracy, precision, recall and F1-score as much as 47.18%, 46.30%, 46.23% and 46.39%, respectively.

## 5. Discussion

In this section, the limitations and the directions of the future research are discussed.

From the experimental results, it is observed that the proposed method can detect adversarial examples while classifying the types of adversarial examples. Although the proposed method showed better binary classification performances than the state-of-the-art defense methods using adversarial example detection architecture, the multi-label classification performance of the proposed method is relatively low compared to the binary classification performances. This is for the following reasons: (1) Inaccuracy in the extraction of adversarial perturbation; and (2) Homogeneity that decreases with the increasing number of adversarial examples. In other words, the high accuracy of adversarial perturbation extraction can help characterize the adversarial perturbation of each adversarial example. The high homogeneity can help the clustering model to match each cluster to each type of adversarial example. Therefore, there are several possible ways to improve the multi-label classification performance of the proposed method: (1) Designing a denoising method specialized for the extraction of adversarial perturbation, rather than the simple application of general denoising techniques, i.e., binary filter and median filter; (2) Applying techniques to improve the Homogeneity such as multi-stage clustering [32,33], growing self-organizing maps [34], and the fractional derivatives [35]. We will leave those kinds of improvements for future work.

In addition, the proposed method can be extended to large-scale application datasets such as the ImageNet dataset and the malware dataset by reflecting some considerations, such as the complexity of high-resolution data and the structured characteristics.

## 6. Conclusions

Adversarial example detection architecture has been actively studied because it can defend adversarial examples without decreasing the accuracy for the legitimate input data. While the previous methods belonging to adversarial example detection architecture can only detect whether adversarial examples have occurred, they cannot know which adversarial example has occurred. In this paper, we propose an advanced defense method using adversarial example detection architecture, which not only detects adversarial examples but also classifies the types of adversarial examples. Specifically, after extracting key features from adversarial perturbation, the proposed method feeds the key features into a clustering model. From evaluation results under various experimental conditions, we showed that the proposed method provided good clustering performance for the MNIST dataset and the CIFAR-10 dataset, respectively. We also observed that the proposed method showed better classification accuracy than recent defense methods using adversarial example detection architecture. From such results, we believe that the proposed method can be actively used to design new defense architecture, analyze the characteristics of adversarial examples, and improve the existing defense methods.

## Figures and Tables

**Table 1:** Summary of the recent defense methods using adversarial example detection architecture. Most of the methods cannot classify the input data into multiple classes of data.

Method	References	Binary Classification	Multi-Label Classification
Signature-based Detector	S. Dathathri et al.’s method [20]	✓	✗
	G. Fidel et al.’s method [21]	✓	✗
Subnetwork-based Detector	Metzen et al.’s method [22]	✓	✗
	Jiajun Lu et al.’s method [23]	✓	✗
GAN-based Detector	Cowboy [24]	✓	✗
	Fangchao Yu et al.’s method [25]	✓	✗
Squeezer-based Detector	Feature Squeezing [16]	✓	✗
	Yu Zheng et al.’s method [26]	✓	✗
LSTM-based Detector	Carrara et al.’s method [15]	✓	✗
	Wang et al.’s method [18]	✓	✗
GNN-based Detector	Abusnaina et al.’s method [17]	✓	✗
Clustering model-based Detector	Proposed method	✓	✓

**Table 2:** Ablationanalysis results of the proposed method.

Dataset	Method	Homogeneity	Completeness	V-Measure	Average
MNIST	Adversarial example + k-means (Step 3)	0.247	0.252	0.249	0.249
	Adversarial perturbation + k-means (Step 1 + Step 3)	0.601	0.654	0.626	0.627
	Adversarial perturbation + LDA + k-means (Step 1 + Step 2 + Step 3)	0.826	0.903	0.863	0.864
CIFAR-10	Adversarial example + k-means (Step 3)	0.092	0.174	0.120	0.128
	Adversarial perturbation + k-means (Step 1 + Step 3)	0.343	0.416	0.375	0.378
	Adversarial perturbation + LDA + k-means (Step 1 + Step 2 + Step 3)	0.676	0.728	0.701	0.701

**Table 3:** Experimental results of the proposed method with the various combinations of dimensionality reduction methods and clustering methods.

Dataset	Dimensionality Reduction Method	Clustering Method	Homogeneity	Completeness	V-Measure	Average
MNIST	PCA	K-means	0.734	0.809	0.770	0.771
		DBSCAN	0.783	0.745	0.764	0.764
	LDA	K-means	0.826	0.903	0.863	0.864
		DBSCAN	0.814	0.919	0.863	0.865
CIFAR-10	PCA	K-means	0.645	0.467	0.646	0.646
		DBSCAN	0.296	0.811	0.434	0.513
	LDA	K-means	0.676	0.728	0.701	0.701
		DBSCAN	0.594	0.854	0.701	0.716

**Table 4:** Comparison results for binary classification with Carrara et al.’s method and Feature Squeezing.

Dataset	Method	Binary Classification			
		Accuracy	Precision	Recall	F1-Score
MNIST	Carrara et al.’s method (LSTM + Euclidean)	71.67% ± 0.24	68.48% ± 0.32	79.35% ± 0.28	73.52% ± 0.27
	Carrara et al.’s method (LSTM + cosine)	70.50% ± 0.52	67.18% ± 0.43	78.34% ± 0.48	72.28% ± 0.46
	Carrara et al.’s method (MLP + Euclidean)	70.18% ± 0.37	67.29% ± 0.34	77.53% ± 0.36	72.13% ± 0.32
	Carrara et al.’s method (MLP + cosine)	69.89% ± 0.64	67.02% ± 0.52	77.24% ± 0.36	71.69% ± 0.36
	Feature Squeezing (Binary filter + $L_{1}$ distance)	94.22% ± 0.21	95.13% ± 0.21	93.20% ± 0.22	94.18% ± 0.21
	Feature Squeezing (Binary filter + $L_{2}$ distance)	94.21% ± 0.23	95.11% ± 0.19	93.30% ± 0.27	94.20% ± 0.20
	Feature Squeezing (Binary filter + *K*-*L* diversity)	95.14% ± 0.20	95.57% ± 0.23	94.45% ± 0.24	94.22% ± 0.25
	proposed method (LDA + DBSCAN)	99.99% ± 0.01	99.99% ± 0.01	99.97% ± 0.05	99.98% ± 0.02
CIFAR-10	Carrara et al.’s method (LSTM + Euclidean)	79.62% ± 0.73	73.58% ± 0.76	91.50% ± 0.67	81.64% ± 0.63
	Carrara et al.’s method (LSTM + cosine)	68.65% ± 1.07	64.68% ± 1.13	78.97% ± 1.11	71.33% ± 1.21
	Carrara et al.’s method (MLP + Euclidean)	77.53% ± 0.86	72.68% ± 0.94	86.70% ± 0.93	79.07% ± 0.87
	Carrara et al.’s method (MLP + cosine)	79.09% ± 1.38	74.99% ± 1.36	85.97% ± 1.39	80.09% ± 1.39
	Feature Squeezing (Binary filter + $L_{1}$ distance)	50.76% ± 0.76	50.31% ± 0.77	49.45% ± 0.82	49.91% ± 0.77
	Feature Squeezing (Binary filter + $L_{2}$ distance)	53.41% ± 0.79	53.12% ± 0.82	51.49% ± 0.87	52.29% ± 0.85
	Feature Squeezing (Binary filter + *K*-*L* diversity)	50.85% ± 0.80	50.48% ± 0.81	49.76% ± 0.84	49.93% ± 0.83
	proposed method (LDA + DBSCAN)	81.32% ± 0.33	82.25% ± 0.32	83.08% ± 0.39	83.17% ± 0.38

**Table 5:** Experimental results for multi-label classification with Carrara et al.’s method and Feature Squeezing.

Dataset	Multi-Label Classification			
	Accuracy	Precision	Recall	F1-Score
MNIST	66.33% ± 2.07	66.29% ± 2.13	66.66% ± 2.20	66.56% ± 2.13
CIFAR-10	47.18% ± 4.31	46.30% ± 4.37	46.23% ± 4.49	46.39% ± 4.46

## Data Availability

The data presented in this study are openly available in reference numbers [27,29].
